# Immune remodeling of tumor-draining lymph nodes: mechanistic determinants of checkpoint blockade responsiveness

**DOI:** 10.3389/fimmu.2026.1963773

**Published:** 2026-09-17

**Authors:** Caiyan Jia, Shuxuan Li, Muhua Chen, Wuxia Yang, Yanqi Song, Aidi Wang, Baoshan Liu

**Affiliations:** Department of Traditional Chinese Medicine, Tianjin Medical University General Hospital, Tianjin, China

**Keywords:** dendritic cell cross-presentation, immune checkpoint blockade, lymph node stromal remodeling, TPEx, tumor-draining lymph nodes

## Abstract

Remodeling of tumor-draining lymph nodes (tdLNs) can precede overt nodal metastasis and has emerged as a determinant of immune checkpoint blockade (ICB) responsiveness. Sustained lymphatic delivery of tumor-derived antigens, extracellular vesicles, cytokines, and metabolites perturbs stromal and vascular programs, including lymphatic expansion, high endothelial venule dysfunction, and disruption of fibroblastic reticular cell (FRC) networks, with downstream impairment of antigen presentation and T-cell priming. Metabolites such as lactate and adenosine further promote tolerogenic antigen-presenting cell states and increase the activation requirements of tumor-reactive T cells. These changes are predicted to reduce the availability of TCF1^+^ progenitor-exhausted CD8^+^ T cells (Tpex), an ICB-responsive reservoir that sustains clonal expansion during PD-1/PD-L1 blockade. The Review links tdLN structural, cellular, and metabolic remodeling to ICB response and resistance, highlights reinforcement of regulatory circuits after metastatic colonization, and discusses translational strategies that incorporate tdLN functional state into therapeutic design, including restoration of type 1 conventional DC (cDC1) competence, metabolic co-targeting, neoadjuvant sequencing, adoptive use of tdLN-derived T cells, and biomarker-guided planning of local therapies.

## 1 Introduction

Tumor-draining lymph nodes (tdLNs) begin changing well before metastases appear (1–3). Unlike the acute metabolic collapse seen in late-stage tumors, tdLN dysfunction develops gradually. Continuous tumor signals drive this process by disrupting architecture, reprogramming resident cells, and rewiring metabolism (4, 5). These changes alter how antigens are presented, how T cells prime, and whether precursor pools persist (6–9). These changes shape the quality of antitumor immunity and influence whether checkpoint blockade can sustain clonal expansion (6, 7, 10).

Lymph nodes sample and integrate signals from peripheral tissues through two coordinated routes. Dendritic cells (DCs) carrying antigens arrive via afferent lymphatics; naïve T and B cells enter through high endothelial venules (HEVs), setting up antigen-specific priming in spatially organized zones (11, 12). In cancer, this system faces persistent exposure to tumor-derived material: antigens, extracellular vesicles (EVs), cytokines, and metabolites flow in continuously, remodeling stromal scaffolds and disrupting vascular programs (4, 5). Over time, this chronic input raises activation thresholds and promotes immunosuppressive nodal remodeling (8).

The link between nodal involvement and distant metastasis has guided surgical practice for decades (2, 13–15). However, growing evidence indicates that intact tdLNs are required for effective checkpoint blockade. Antitumor responses start and are maintained in tdLNs, sustained by Tpex cells that continuously supply intratumoral effectors during anti-PD-1 therapy (6, 7, 10). Removing nodes thus depletes the upstream reservoir—clearing local disease while potentially sacrificing long-term immune control (8, 10). Supporting this concept, systematic tdLN dissection in early-stage lung adenocarcinoma was associated with greater postoperative declines in circulating Th1-like CD4^+^ T cells and worse oncologic outcomes (16).

A key distinction separates tdLNs from the tumor microenvironment (TME). Lactate, oxidized lipids, kynurenine, and adenosine reach tdLNs through lymph, but at levels too low to starve T cells competitively (17–20). Instead, they act as signals—raising activation thresholds and biasing differentiation. This suggests that resistance may emerge from impaired immune generation upstream in tdLNs rather than exclusively within tumors (21, 22).

This Review focuses on three interconnected ways tdLNs remodel under tumor influence, all of which shape therapeutic outcomes. Structurally, lymphatics expand, HEVs fail, and fibroblastic reticular cell (FRC) networks collapse. At the cellular level, DCs lose maturation capacity, macrophages polarize, and Tregs accumulate. Metabolically, tumor-derived molecules reset activation thresholds and redirect T-cell fates. We discuss how these remodeling programs interact, influence reversibility, and shape strategies to preserve or restore upstream immune function.

## 2 Structural organization and immune architecture of tdLNs

tdLNs constitute the first organized immune structure encountering tumor-derived material. Antigen transport via lymphatics, stromal topology, and immune cell positioning collectively determine whether tumor-specific responses initiate or deviate. Beyond passive conduction, tdLNs actively generate immune output.

### 2.1 Lymphatic and vascular infrastructure

Antigen delivery and lymphocyte recruitment into tdLNs are coordinated through two anatomically distinct trafficking systems: afferent lymphatics and blood vascular networks. Lymphatic endothelial cells (LECs) lining the subcapsular sinus and medullary regions secrete CCL21, establishing gradients that direct CCR7^+^ DCs from the draining tissue toward the paracortical T-cell zone (11, 12). Concurrently, HEVs mediate naïve and central memory lymphocyte entry via peripheral node addressin (PNAd)–L-selectin adhesion and CCL19/CCL21-dependent transmigration, sustaining a continuous precursor supply (23, 24). The functional integrity of both systems is required for efficient antigen–lymphocyte colocalization.

Beyond transport, LECs actively contribute to immune regulation. Under homeostatic conditions, LECs and FRCs present peripheral tissue antigens on major histocompatibility complex (MHC) class II, contributing to deletion of autoreactive clones and maintenance of peripheral tolerance (25–27). LECs additionally support the retention of subcapsular sinus macrophages (SSMs), which intercept lymph-borne particulate material—including tumor-derived EVs—prior to deeper cortical penetration (5, 28). LEC dysfunction arising from sustained tumor drainage therefore disrupts both antigen entry kinetics and the first-pass filtration function of the subcapsular sinus.

Beyond vascular and lymphatic conduits, tdLNs are gated by direct neural input. Sympathetic and sensory fibers innervate the parenchyma, where adrenergic tone regulates lymphocyte residency and antitumor immunity. β2-Adrenergic signaling, for example, forms CCR7/CXCR4 receptor complexes that retain lymphocytes within the node while curtailing effector programs (29). More strikingly, exhausted CD8^+^ T cells congregate around sympathetic nerve terminals under ADRB1 drive, and this perineural sequestration is reversed by adrenergic blockade with concomitant recovery of cytotoxicity (30). Whether tumor progression actively reshapes nodal sympathetic innervation—effectively adding a neural layer of immunosuppression to the stromal and vascular remodeling outlined above—remains to be determined, but the clinical availability of β-blockers makes this an immediately actionable question for ICB combination strategies (31).

### 2.2 Stromal networks and immune cell niches

FRCs constitute the reticular scaffold of the lymph node and are functionally non-redundant across subsets. T zone FRCs produce IL-7, CCL19, and CCL21 at concentrations sufficient to sustain naïve T-cell survival and recirculation in the absence of antigen (32, 33). Perivascular FRCs facilitate DC positioning and direct soluble antigen through conduit networks. Medullary FRCs regulate lymphocyte egress through sphingosine-1-phosphate (S1P) gradients (32, 33). This functional compartmentalization creates topographically distinct priming niches. Selective disruption of individual FRC subsets by tumor-derived signals can therefore rewire the spatial organization of immune priming.

SSMs constitute the primary cellular checkpoint for lymph-borne antigen. Positioned at the subcapsular sinus, they capture particulate antigen and tumor-derived EVs ahead of cortical entry. CD169^+^ SSMs can cross-present dead-cell-associated tumor antigens to naïve CD8^+^ T cells, supporting cytotoxic priming in murine models (13). Medullary macrophages, responding to distinct local signals, are prone to tolerogenic polarization (13). The net immunological output of antigen arrival is therefore partly determined by the relative representation of these macrophage subsets at the initial antigen encounter site.

For CD8^+^ T-cell priming, type 1 conventional DCs (cDC1s) are non-redundant. Nodal homing via CCR7 and CD4^+^ T-cell licensing through CD40–CD40L are both required for productive cytotoxic differentiation (34, 35). Nodal cDC1 abundance and maturation state predict ICB responsiveness (6, 36), and deficits in cDC1 maturation within tdLNs have been documented to precede systemic T-cell dysfunction in multiple tumor models (8, 9)—indicating that antigen-presentation failure at the nodal level represents an upstream determinant of therapeutic outcome.

### 2.3 Baseline immune cell composition

Under homeostatic conditions, tdLNs maintain Tpex pools that sustain systemic and intratumoral immune responses. Following antigen-driven activation, CD8^+^ T cells exit the node via S1PR1-dependent egress and reseed peripheral tissue. Pharmacological blockade of this egress with fingolimod (FTY720) substantially impairs antitumor efficacy in murine tumor models regardless of intratumoral T-cell status, confirming that continuous nodal export of primed effectors is rate-limiting for systemic tumor control (10). Single-cell RNA sequencing and T cell receptor (TCR) lineage tracing have established that tdLNs harbor Tpex cells with retained proliferative potential and responsiveness to ICB (6, 7). These cells are metabolically and transcriptionally distinct from terminally exhausted populations—a distinction with direct consequences for their maintenance within nodal niches and their responsiveness to ICB. Human TCR clonotype analyses confirm shared clonal origins between tdLN Tpex and matched intratumoral exhausted CD8^+^ T-cell populations (6), establishing a direct lineage relationship and indicating that nodal priming quality determines the functional composition of the intratumoral T-cell compartment.

CD4^+^ T-cell subsets exert additional modulatory influence. Conventional helper CD4+ T cells support cDC1 activation and memory CD8^+^ T-cell formation (34). During tumor progression, however, tdLNs become increasingly tumor-conditioned, with cumulative defects in antigen-presenting cell activation and enrichment of suppressive cues. These changes can plausibly shift germinal center dynamics by favoring regulatory programs (including Tfr differentiation/expansion) and limiting effective Tfh help. Accordingly, changes in the T follicular helper (Tfh)-to-T follicular regulatory (Tfr) balance may alter germinal center output and the quality of tumor-reactive antibody responses—supporting antitumor humoral immunity in some settings while, in others, facilitating antibody programs associated with immune tolerance and nodal metastasis (37).

## 3 Tumor-induced remodeling programs

Structural remodeling of **tdLNs** begins before overt immune dysfunction or metastatic colonization becomes clinically evident. Tumor-derived signals reshape tdLNs through temporally overlapping processes: initial vascular conditioning, progressive deterioration of stromal and cellular networks, and sustained metabolic perturbations that collectively elevate activation thresholds.

### 3.1 Early-stage remodeling: pre-metastatic conditioning

Nodal remodeling begins before tumor cells become histologically detectable within lymph node parenchyma. Continuous lymphatic drainage delivers tumor-derived antigens, EVs, cytokines, and soluble mediators into draining basins. Melanoma and breast cancer models demonstrate that this exposure triggers lymphatic sinus dilation, extracellular matrix reorganization, and morphological changes in high endothelial venules well before overt metastasis (1, 2, 4, 5, 38). Sentinel lymph node analyses from patients lacking histologically confirmed metastasis reveal similar vascular and stromal abnormalities (3, 39).

Murine models (B16 melanoma and MC38 colorectal cancer) reveal a consistent timeline. During early phases, tumor-derived vascular endothelial growth factor C (VEGF-C) engages VEGF receptor 3 (VEGFR3) on LECs, transducing downstream PI3K-AKT and ERK1/2 signaling to drive lymphatic sinus dilation and remodeling (40, 41). Concurrently, melanoma-derived small EVs further promote lymphangiogenesis via nerve growth factor receptor (NGFR)-dependent pathways (4), while vesicle-associated integrins govern organotropic pre-metastatic niche formation (42). As the disease progresses, structural destabilization accelerates: high endothelial venules lose PNAd expression and L-selectin binding capacity (24, 40, 43), and FRC networks contract within T zones (44). These structural alterations translate directly to human disease; melanoma and oral squamous cell carcinoma patients lacking nodal metastases already exhibit pronounced vascular expansion and increased lymphatic vessel density in sentinel nodes (3, 45), confirming an active preparatory program.

Parallel to structural remodeling, pre-metastatic conditioning actively installs a tolerogenic microenvironment. Tumor-derived EVs accumulate rapidly in CD169^+^ subcapsular sinus macrophages (4, 41), engaging TLR–MyD88 signaling to trigger NF-κB–dependent transcription of suppressive cytokines, including IL-10 and TGF-β (46). In tandem, LECs upregulate CD39 and CD73 to initiate adenosine-mediated immunosuppression (47), while homeostatic CCL21 gradients deteriorate (42, 43). Importantly, this early niche conditioning directly disables adaptive antitumor immunity: disrupted chemokine guidance impairs the trafficking and positioning of CCR7^+^ migratory dendritic cells essential for CD8^+^ T-cell cross-priming (48), while remodeled LECs and stromal networks actively induce antigen-specific tolerance (49). Ultimately, the convergence of altered sinus architecture, aberrant cargo distribution across nodal microdomains (32, 45, 50), and pervasive stromal suppression progressively cripples the capacity of tdLNs to prime effective antitumor responses, establishing an immunologically privileged pre-metastatic niche prior to overt nodal colonization (51) (**Figure 1**).

**Figure 1:**
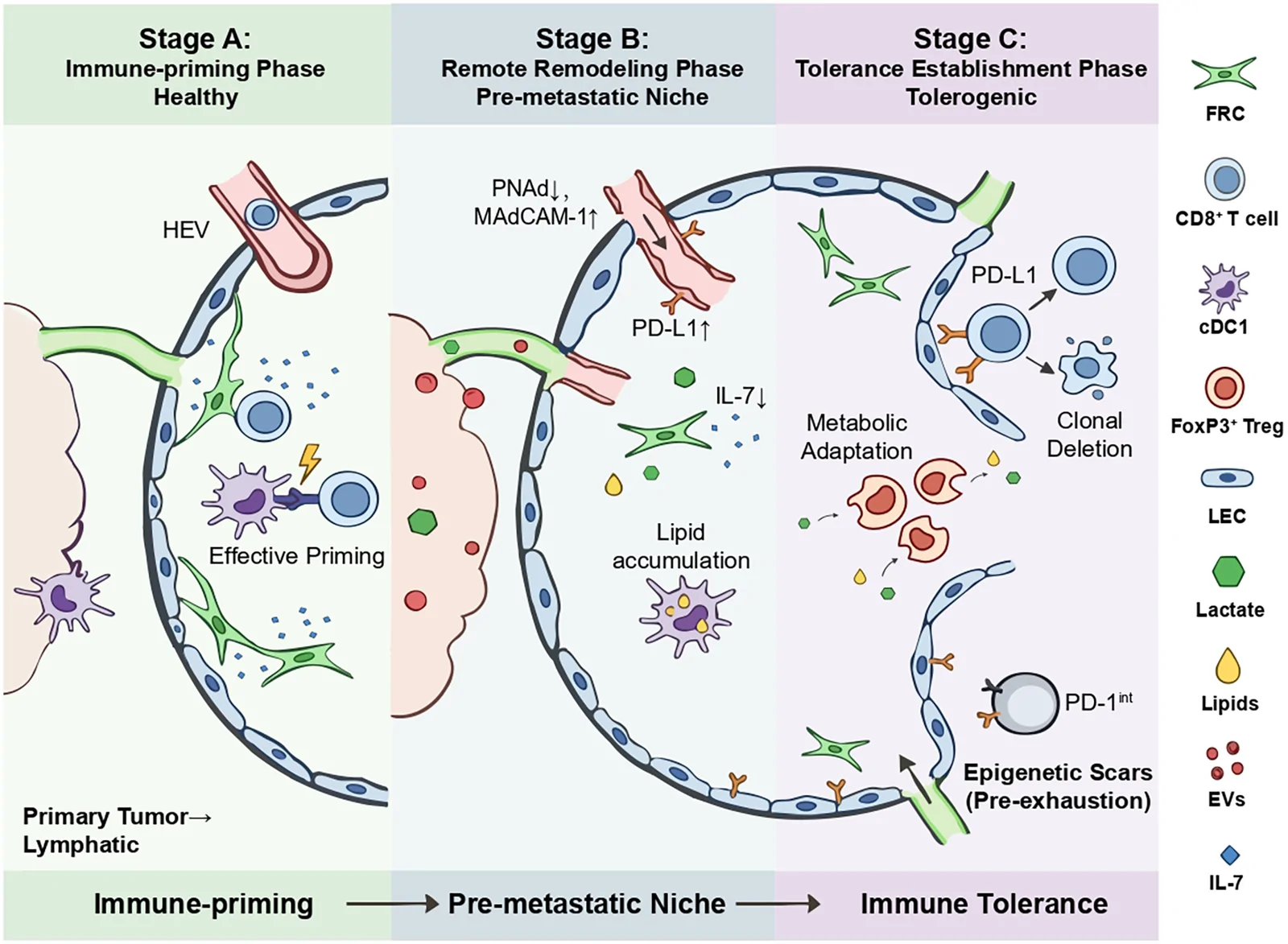
Tumor-draining lymph nodes transition from immune-competent priming hubs (left) to immunosuppressive pre-metastatic niches (right). Key structural changes include lymphatic expansion, HEV dysfunction, FRC network collapse, and regulatory cell accumulation, culminating in reduced ICB responsiveness.

### 3.2 Progressive stromal and cellular dysfunction

Vascular alterations coincide with broader disruption of stromal infrastructure and immune cell composition. Tumor drainage systematically reprograms FRC networks, which ordinarily maintain conduit systems that distribute antigen and coordinate DC–T cell encounters (32, 50, 52, 53). Murine tumor models document shifts in extracellular matrix composition, chemokine secretion patterns, and leukocyte positioning as components of comprehensive stromal transformation (47). Human breast cancer specimens confirm perivascular stromal dysregulation and disrupted CCL21 expression (39).

Stromal injury propagates to adaptive immunity through defined metabolic routes. Tumor-derived lactate delivered via tumor-draining lymph disrupts stromal homeostasis and shifts dendritic cells toward tolerogenic programs. Antigen delivery and productive priming thereby become uncoupled—antigen continues to drain, yet priming output and quality progressively decline and become increasingly biased toward tolerance.

Beyond this metabolic perturbation, tumor-conditioned FRCs actively orchestrate the recruitment of suppressive myeloid populations. Upregulation of CCL2 and CCL7 by FRCs drives the influx of CCR2^+^ monocytes into tdLNs, where these cells acquire a PD-L1hi, iNOS-expressing phenotype that directly constrains T-cell proliferation within nodal niches, establishing a TLR4-dependent fibroblast–monocyte suppressive axis operating upstream of overt metastatic colonization (54). Sustaining this circuit, constitutive STAT3 phosphorylation governs the expansion and immunosuppressive function of these monocytic myeloid-derived suppressor cells (MDSCs) while blocking their terminal differentiation into immunocompetent DCs and macrophages (55), thereby locking the nodal microenvironment into a chronic inhibitory state that precedes intratumoral T-cell exhaustion.

Immune cell composition shifts in parallel. FoxP3^+^ regulatory T cell (Treg) expansion within tdLNs correlates with nodal involvement and adverse outcomes across multiple tumor types (56–58). Functionally, nodal Tregs amplify local suppressive circuits, with their inhibitory influence extending to both CD4^+^ helper and CD8^+^ cytotoxic priming axes (59). Myeloid populations undergo comparable reorientation. DC activation in sentinel nodes deteriorates before systemic suppression becomes apparent (8, 9). PD-L1 expression on nodal DCs constrains local priming independently of intratumoral exhaustion, and blocking this pathway restores proliferative responses ex vivo (8). SSMs intercept extracellular vesicle cargo and modulate antigen propagation, while context-dependent polarization favors tolerogenic microdomains (13).

Tumor-mediated nodal subversion reaches well beyond the DC–T cell axis to engulf both humoral and granulocytic compartments. Sentinel and tdLNs from cancer patients frequently lack active germinal centers, harboring instead quiescent memory B cells that share clonal ancestry with intratumoral memory B and plasma cells—a lineage continuity reminiscent of the tumor-infiltrating CD8^+^ T-cell repertoire (60). This humoral silencing coincides with an accumulation of DC-SIGN+ macrophages, whose abundance inversely correlates with germinal center B cells and suggests active myeloid suppression of nodal antibody responses (61). Neutrophils undergo a corresponding shift: although early nodal neutrophils can mount antitumor activity, disease progression prompts phenotypic divergence from their circulating counterparts (62, 63). Such multi-lineage deregulation highlights that pre-metastatic tdLN dysfunction fundamentally disables both cellular and humoral arms of adaptive immunity, thereby eroding the systemic substrate required for effective immune checkpoint blockade.

As tumor burden increases and metastatic colonization occurs, tolerance programs intensify and Tpex dynamics deteriorate (6, 13). This progression unfolds along a continuum rather than through binary transition. Early-conditioned nodes retain partial priming competence, whereas metastatic nodes accumulate compounded structural and regulatory constraints resistant to therapeutic reprogramming (6, 64).

### 3.3 Metabolic gatekeeping: reshaping activation thresholds

Tumor-derived metabolites arrive before malignant cells colonize the node, delivered continuously via lymphatic drainage (2, 4, 13, 65). Compared with the hypoxic, nutrient-restricted tumor bed, tdLNs are generally less constrained by oxygen and glucose availability and are therefore not dominated by classic nutrient-competition mechanisms (66). Instead, tumor-derived metabolites are more likely to act as signaling cues that raise activation thresholds and reprogram stromal and antigen-presenting cell function, ultimately constraining priming efficiency and the stability of precursor compartments.

These gates do not abolish priming categorically but progressively constrain response quality, durability, and lineage commitment.

Lactate and extracellular acidification emerge as prominent candidates in tumor-associated immune dysfunction (53, 67, 68). Highly glycolytic tumors export lactate through monocarboxylate transporters (MCTs) (69), delivering lactate-enriched lymph to tdLNs. Effector CD8^+^ T cells and natural killer (NK) cells show marked sensitivity to lactate, whereas Tregs maintain metabolic resilience under low-glucose, high-lactate conditions (17, 70–72). This differential sensitivity suggests that lactate exposure selectively skews differentiation trajectories rather than eliminating nodal immunity outright.

Mechanistically, lactate exposure within FRC niches impairs mitochondrial function and suppresses IL-7 transcription (73); because IL-7 sustains naïve and precursor CD8^+^ T-cell pools, this decline destabilizes the Tpex compartment even as antigen drainage continues. In parallel, lactate signals through SREBP2-dependent lipogenic programs to push dendritic cells toward tolerogenic maturation, directly impairing cross-presentation capacity (74). Histone lactylation demonstrates that such metabolic exposure can, in principle, be inscribed into chromatin (75); whether lactate concentrations in human tdLNs ever reach the levels required for durable epigenetic reprogramming remains unknown. Functionally, lactate is best framed as a gate on activation intensity and lineage bias.

Lipid transport constitutes an intrinsic feature of lymphatic physiology (65), rendering lipid perturbations particularly relevant to tdLN dysfunction. Tumors deliver lipid-rich EVs and lipoprotein-like particles into draining lymph (4, 38, 42, 65, 76), conditioning stromal and myeloid compartments. Lipid accumulation in DCs impairs cross-presentation, induces oxidative stress, and promotes endoplasmic reticulum stress. Within tumors, oxidized lipid uptake also drives metabolic dysfunction in effector CD8^+^ T cells while sparing regulatory populations (77–79). Beyond direct functional impairment, altered lipid metabolism may further reshape transcriptional programs through acetyl-CoA availability and histone acetylation dynamics (80, 81), linking metabolic flux to chromatin regulation. Although direct evidence in human tdLNs remains limited, lipid remodeling remains a plausible mechanism affecting antigen presentation and T-cell fate.

IDO1 expression in tdLNs gained attention through studies documenting local tryptophan catabolism within regional immune compartments (19, 82, 83). Recent analyses of human tdLNs identify immunosuppressive transcriptional signatures associated with poor clinical outcomes, with IDO1-related pathways featuring prominently (6, 56, 84). These findings position tryptophan metabolism as a nodal constraint on antitumor priming (85). Tryptophan depletion and kynurenine generation operate through parallel mechanisms: substrate restriction limits CD8^+^ T-cell proliferation, while kynurenine signaling via the aryl hydrocarbon receptor promotes regulatory CD4^+^ T cell differentiation (83, 85–87). Myeloid-derived suppressor cells expressing IDO1 further reinforce this axis within nodal compartments (87).

Arginine restriction adds another layer of metabolic constraint. Arginase activity within suppressive myeloid populations depletes local arginine, impairing T-cell proliferation and effector differentiation partly through TCRζ downregulation (88, 89). Although direct arginine measurements within tdLNs remain limited, studies in tumor-bearing hosts support a role for arginine deprivation in promoting dysfunctional immunity (87, 90).

One-carbon metabolism may further influence precursor maintenance under metabolic stress (81, 91). The folate and methionine cycles provide nucleotides for proliferation and methyl donors for epigenetic regulation, while altered methionine availability can suppress effector-associated transcriptional programs (80, 81). Whether nodal Tpex populations display unique dependencies on one-carbon pathways remains unclear, but metabolic disruption within these pathways may constrain precursor maintenance and differentiation plasticity without abolishing priming.

The ATP-to-adenosine axis integrates tissue stress into immunosuppressive signaling (45, 92). Transient extracellular ATP release can activate antigen-presenting cells, but sustained upregulation of CD39 and CD73 on LECs, Tregs, and suppressive myeloid populations converts ATP into adenosine (45, 93, 94). Adenosine signals through A2A receptors (A2AR), elevating intracellular cyclic adenosine monophosphate (cAMP) and dampening proximal TCR signaling, thereby raising activation thresholds for T cells attempting to prime within nodes (95, 96). In DCs, adenosine induces arginase 2 and IDO1 expression (97, 98), linking purinergic signaling to amino acid restriction pathways. Spatially restricted adenosine-rich microdomains—at antigen-presenting cell–T cell interfaces or lymphatic sinus borders—may suppress priming locally without saturating the entire node (93, 99). Hypoxia and lactate independently upregulate CD39 and CD73 through hypoxia-inducible factor-1α (HIF-1α)-dependent mechanisms (96, 100, 101), positioning purinergic signaling as a convergence point for multiple metabolic insults. Collectively, these metabolic programs do not simply suppress immunity; they progressively recalibrate the activation threshold required for productive priming, thereby shifting tdLNs from permissive immune niches toward metabolically gated states of tolerance (**Figure 2**).

**Figure 2:**
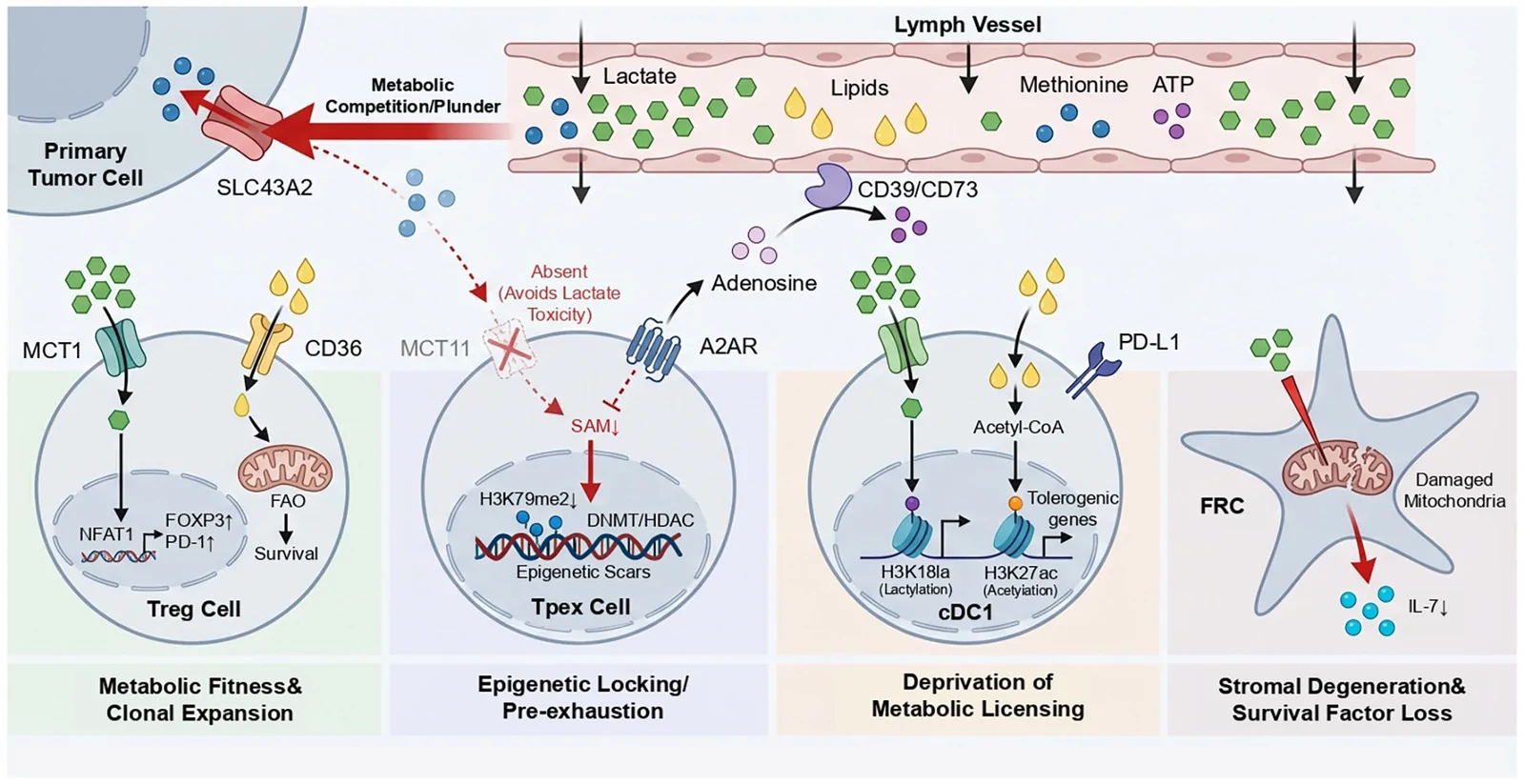
Metabolic–epigenetic coupling drives immune reprogramming within tumor-draining lymph nodes. Tumor-derived metabolites reprogram stromal and immune cells in tdLNs through coupled metabolic and epigenetic mechanisms. Lactate, lipids, and amino acid–derived signals impair dendritic cell function, disrupt stromal support, and promote regulatory and exhausted T-cell states, collectively suppressing effective antitumor immunity.

## 4 T cell differentiation trajectories in remodeled tdLNs

By the time a T cell enters a tumor, much of its fate has already been determined—not by what it encounters inside the tumor, but by what happened to it in **tdLNs**. The priming quality, metabolic configuration, and chromatin state carried into the TME are largely shaped by the nodal environment in which T cells were initially programmed. In a remodeled node, each of these parameters is compromised before any direct intratumoral encounter occurs.

### 4.1 Tpex dynamics: nodal origin, spatial maintenance, and metabolic vulnerability

PD-1/PD-L1 blockade efficacy depends substantially on the preservation of Tpex cells within tdLNs. Murine lineage-tracing experiments establish that nodal Tpex cells clonally seed intratumoral exhausted populations (7, 22, 23). Epigenomic profiling demonstrates that, despite chronic antigen exposure, nodal Tpex retain chromatin accessibility permissive for proliferative renewal (102, 103). A functional distinction between nodal and intratumoral Tpex compartments has been established. Intratumoral Tpex cells progressively acquire tissue-residency programs associated with reduced migratory capacity and diminished responsiveness to ICB (104). By contrast, nodal Tpex maintain stem-like programs supported by MYB-dependent transcriptional networks. MYB regulates trafficking-associated programs, including CCR7/CXCR5 expression; MYB deficiency reduces antigen-specific CD8^+^ T-cell accumulation in lymph nodes (105).

Beyond transcriptional regulation, spatial organization within tdLNs contributes to Tpex maintenance. Tpex cells preferentially localize within IL-7hi FRC niches in T-cell zones (106), where IL-7 signaling supports survival and limits premature differentiation (33). Tumor-induced disruption of FRC networks therefore directly contributes to precursor depletion, suggesting that stromal restoration strategies may complement T-cell-directed interventions.

Metabolically, Tpex cells preserve mitochondrial integrity and limit glycolysis relative to terminally exhausted populations (107–111). P4HA1 disrupts mitochondrial metabolism and selectively depletes nodal Tpex cells; its inhibition restores precursor fitness and improves responses to ICB and adoptive therapy in preclinical systems (112).

### 4.2 Exhaustion priming in chronically conditioned nodes

T-cell exhaustion is not initiated exclusively within tumor tissue. Sustained TCR signaling in tdLNs exposed to chronic antigen drives nuclear factor of activated T cells (NFAT)-dependent recruitment of TOX and NR4A transcription factors, progressively restricting chromatin accessibility and developmental flexibility (107, 108, 113–115). Nevertheless, chronic antigen exposure is not required for all forms of nodal T-cell dysfunction, as defective CD8^+^ T-cell priming has also been demonstrated in pre-metastatic tdLNs independently of chronic antigenic stimulation (116).

Single-cell epigenomic analyses distinguish two functionally discrete Tpex substates. Tpex-early cells (TOX^lo^ TCF1^hi,^ reversible chromatin) respond to PD-1 monotherapy and predominate in pre-metastatic nodes. Tpex-early cells may display intermediate exhaustion features, including PD-1int states, while retaining reversible chromatin and PD-1 responsiveness. Tpex-late cells (TOX^hi^ TCF1^int^, partially fixed chromatin) require combination strategies incorporating epigenetic modulation, and their accumulation in chronically conditioned or metastatic nodes correlates inversely with ICB responsiveness (6, 56, 102, 117). This substrate shift provides a mechanistic basis for the superior efficacy of neoadjuvant over adjuvant ICB observed across multiple tumor types.

These transcriptional limits are compounded by tumor-conditioned metabolism in the node. Impaired DC cross-presentation, adenosine-mediated dampening of TCR signals, amino-acid shortage, and mitochondrial stress together skew T-cell differentiation, often without extinguishing priming altogether (74, 110, 111, 118, 119).

### 4.3 CD4^+^ regulation of CD8^+^ differentiation in remodeled nodes

CD4^+^ T helper cells license cDC1s through CD40–CD40L interactions, a requirement for sustained cross-presentation and durable CD8^+^ memory formation (34) (120). Disruption of this licensing axis reduces CD8^+^ priming both quantitatively and qualitatively.

FoxP3^+^ Treg accumulation in tdLNs is documented across solid tumor types and correlates with nodal metastasis and adverse prognosis (57, 58, 121). Recent studies show that IL-33 released by CD169^+^ macrophages promotes expansion of ST2^+^ Tregs that migrate to tumors and suppress CD8^+^ T-cell responses; blockade of this axis improves responses to ICB and chemotherapy (122).

Treg metabolic resilience compounds their competitive advantage in conditioned nodes. Tregs exhibit metabolic adaptations, including enhanced glycolytic flexibility and fatty acid oxidation, enabling survival under nutrient-restricted conditions (70–72, 79, 120). As a result, the Treg-to-effector ratio increases through selective effector attrition rather than Treg expansion per se (57, 123). Nodal Tregs additionally induce anergy in tumor-specific CD4^+^ effectors (59), reducing helper signals required for CD8^+^ commitment.

Spatial analyses of human tdLNs identify suppressive niches near lymphatic sinuses where Tregs and tolerogenic DCs co-localize (99, 124). Progressive Tfh/Tfr imbalance and B-cell remodeling shift nodal immune tone over time (13). In metastatic nodes, reduced Tpex frequency and expanded Treg networks consistently co-occur (125), converging to diminish the regenerative capacity on which durable ICB responses depend.

## 5 Mechanistic basis of ICB response and resistance

Resistance to ICB is often framed primarily through tumor-intrinsic or intratumoral immune escape mechanisms. This perspective proves incomplete. Lymph nodes that generate and sustain tumor-reactive T cells shape therapeutic outcomes as profoundly as events within tumors themselves, and variability in nodal state at treatment onset influences clinical trajectories.

### 5.1 Nodal clonal renewal as an ICB requirement

PD-1/PD-L1 blockade is typically framed as reactivating exhausted T cells within tumor tissue. Experimental evidence points to a more distributed mechanism. Preventing lymphocyte egress from tdLNs with FTY720 substantially reduces PD-1 inhibitor efficacy in murine systems, even when intratumoral T-cell numbers remain intact (8, 10). Similarly, elective nodal irradiation dampens systemic immune responses generated by combined radiotherapy and ICB (126), whereas delaying tdLN irradiation preserves therapeutic efficacy in metastatic models (127, 128).

Clonotype analysis exposes the nodal basis of ICB efficacy. In responding patients, 60–80% of tumor-infiltrating T-cell clones originate from tdLNs and undergo peak expansion there during weeks 2–4 post-treatment before migrating to tumor sites; by contrast, non-responders show clonal concordance below 30% (6, 129), with early Tpex pool collapse likely reflecting pre-existing stromal remodeling. The repertoire becomes dominated by TCF1lo terminally exhausted clones that cannot be reinvigorated by PD-1 blockade (6, 130). ICB thus unmasks the quality of nodal priming established weeks to months before treatment initiation. Durable benefit depends substantially on preserving the Tpex compartment (103).

Longitudinal TCR profiling in melanoma and lung cancer patients confirms that CD8^+^ T-cell expansion after PD-1 blockade frequently involves recruitment of new clonotypes rather than simple re-expansion of pre-existing exhausted populations (6, 130, 131). tdLN analyses demonstrate that TCF1^+^ Tpex cells expand during therapy, and their clonal connectivity to tumor-infiltrating effectors provides the mechanistic basis for sustained intratumoral T cell renewal under PD-1 blockade (6, 7). Once nodes become metastatically colonized, this clonal continuity weakens and precursor frequencies decline (6).

### 5.2 Remodeling mechanisms that predict resistance

Primary and acquired resistance may both reflect treatment initiation in lymph nodes already remodeled by chronic tumor-derived signals (64). Sentinel node analyses in breast cancer and melanoma patients reveal impaired DC activation before overt systemic T cell dysfunction becomes detectable (9, 56, 129, 132). Structural remodeling of high endothelial venules reduces naïve T cell recruitment and disrupts DC positioning (39, 40). Under these conditions, antigen continues to drain yet priming efficiency declines. Resistance mechanisms within tdLNs can be stratified according to their dominant biological constraints and relative reversibility.

Resistance within tdLNs can be parsed by its dominant constraint. When DC maturation fails, the block is largely functional and often reversible with innate agonists or checkpoint combinations. Loss of HEVs and collapse of FRC networks—seen as fewer PNAd^+^ vessels—produce a structural barrier that is far harder to restore (24, 40, 44, 47). Once the Tpex pool is depleted and Treg/CD8 ratios rise, the defect becomes clonal: residual priming capacity is minimal, and recovery usually depends on adoptive cell therapy (ACT) rather than ICB alone (56, 133).

This framework explains why neoadjuvant ICB outperforms adjuvant strategies, as it engages a Tpex pool before extensive nodal remodeling (100, 134). In metastatic nodes, coordinated regulatory circuits and dysfunctional T cell states are associated with reduced responsiveness to ICB (56, 128), consistent with impaired precursor renewal capacity (**Figure 3**).

**Figure 3:**
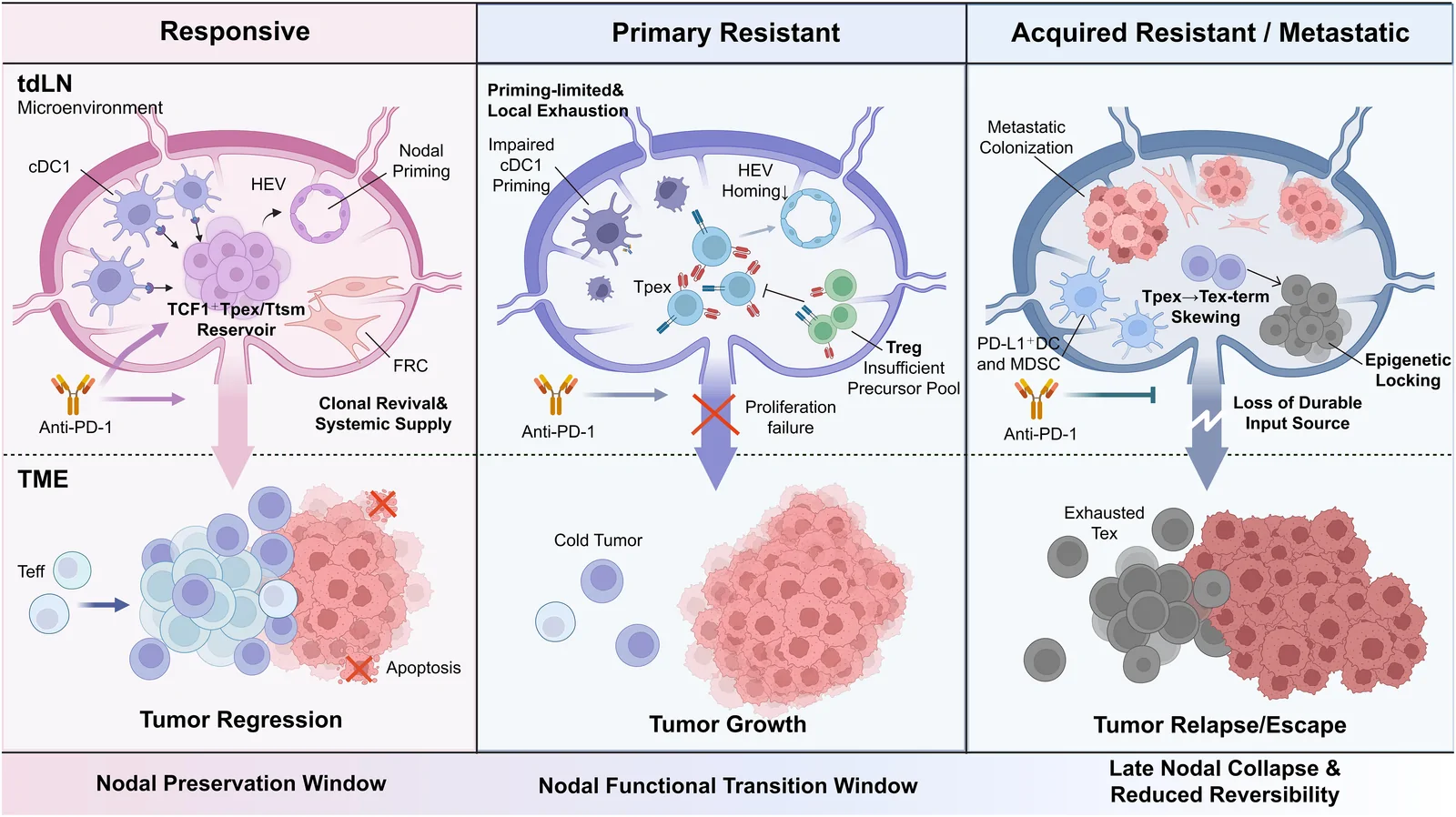
Functional states of tumor-draining lymph nodes determine responsiveness and resistance to immune checkpoint blockade tdLNs exist in distinct functional states that determine ICB outcomes. Immune-competent nodes sustain TCF1^+^ progenitor CD8^+^ T cells and effective priming, partially remodeled nodes exhibit impaired antigen presentation and reduced precursor generation, and advanced nodes show structural collapse and T-cell exhaustion, resulting in primary or acquired resistance to ICB.

### 5.3 Nodal microenvironment versus TME: distinct resistance mechanisms

Acquired resistance unfolds gradually rather than through discrete events. Chronic antigen drainage, repeated suboptimal priming, and sustained metabolite exposure progressively shift Tpex-like states toward fixed dysfunctional trajectories, particularly once exhaustion-associated chromatin programs stabilize (114, 117, 135).

tdLN-based resistance diverges mechanistically from TME-based resistance. In the TME, metabolic stress and nutrient competition, hypoxia-linked programs, and stromal barriers can converge to limit infiltration and drive dysfunction in tumor-reactive T cells at effector sites (22, 67, 111, 114). By contrast, tdLN-based resistance reflects impaired immune generation upstream of the tumor, including metabolite-driven tuning of activation thresholds (for example, lactate-conditioned stromal niches), reduced lymphocyte recruitment due to HEV dysfunction, and failure to sustain Tpex pools during priming (7, 8, 24, 73). Clinically, focal radiation and other intratumoral interventions may be insufficient when tdLN function is compromised, as suggested by studies showing loss of synergy when tdLNs are electively irradiated and preservation of efficacy when tdLN irradiation is delayed (126, 127). Conversely, improving nodal priming without addressing dominant intratumoral constraints may still yield T cells that cannot infiltrate or persist in tumors (22). These considerations favor treatment strategies that preserve or restore tdLN competence while simultaneously reducing key intratumoral barriers (64, 136).

Metastatic tdLN involvement accelerates nodal dysfunction. tdLN colonization induces tumor-immune tolerance and promotes distant dissemination in experimental systems (13). In patients, metastatic nodes exhibit reduced Tpex compartments, altered stromal programs, and expanded regulatory networks relative to non-metastatic nodes (6, 22, 56). Spatial multi-omic analyses identify intensified Treg–myeloid suppressive circuits following metastatic involvement (99, 122). Therapeutic disruption of nodal infrastructure—whether through surgical dissection or radiation field expansion—eliminates the upstream precursor compartment on which sustained ICB efficacy depends. This converts an immune-generative organ into an absent or dysfunctional one, thereby limiting the clonal renewal that distinguishes durable from transient responses (114, 126, 128).

## 6 Translational perspectives

Regional lymph node management has historically been guided by patterns of metastatic dissemination. With immunotherapy integrated into standard oncologic care, therapeutic decisions increasingly extend beyond tumor clearance to include preservation or restoration of immune competence. Conceptually, tdLN-directed interventions can be stratified according to whether they restore antigen presentation, expand or preserve T-cell precursor pools, exploit nodal lymphocyte reservoirs, or guide treatment planning through functional biomarkers. The immunologic state of tdLNs at the time of intervention is therefore a critical determinant of therapeutic efficacy.

### 6.1 Restoring antigen presentation competence

Checkpoint blockade removes inhibitory signaling but does not correct upstream defects in antigen priming. When DC function is compromised within tdLNs, PD-1/PD-L1 blockade amplifies only pre-existing immune capacity.

Flt3L expands cDC1 populations and enhances cross-presentation, synergizing with PD-L1 blockade in preclinical models (137). Innate immune agonists, including TLR and STING ligands, restore DC activation programs and reverse tolerogenic states associated with metabolic conditioning (74, 138). MCT1 inhibition can partially restore DC function by limiting lactate uptake and improving antigen presentation (139).

Vaccination strategies, including neoantigen-based vaccines and lymph node–targeted nanoparticle or mRNA platforms, enhance cross-presentation and CD8^+^ T-cell priming, often synergizing with checkpoint blockade (140–143).

Exogenous DC-based vaccines offer a complementary approach when endogenous antigen presentation in tdLNs is impaired. Antigen-loaded DCs generated ex vivo can migrate to tdLNs and prime antigen-specific T cells, although nodal trafficking depends on the route of administration and remains quantitatively limited (144, 145). DC vaccination can also broaden the repertoire of neoantigen-specific T cells (146), providing a larger pool of tumor-reactive cells that may subsequently respond to checkpoint blockade. A recent phase I study combining intratumoral autologous CD1c^+^/CD141^+^ myeloid DCs with intratumoral ipilimumab, AS01B, and low-dose nivolumab demonstrated feasibility, good tolerability, and durable responses in ICI-refractory melanoma (147). Preclinical data localize this synergy to tdLNs. Randomized trials of DC monotherapy have been negative (148, 149), reinforcing its role as an upstream complement to ICB rather than a stand-alone treatment.

### 6.2 Expanding and protecting Tpex reservoirs

Durable immunotherapy responses depend on the maintenance of Tpex cells, which reside primarily in tdLNs and related lymphoid structures. Higher Tpex abundance correlates with improved clinical outcomes (6, 109, 150–152). Neoadjuvant checkpoint blockade engages precursor pools prior to extensive nodal remodeling, resulting in improved outcomes compared with adjuvant therapy in melanoma and other malignancies (153–155). Early intervention preserves stromal and vascular support required for Tpex maintenance and promotes systemic clonal expansion from nodal reservoirs (156, 157).

Metabolic and cytokine-based strategies may support the Tpex compartment. P4HA1 inhibition restores mitochondrial fitness and expands Tpex (115). IL-7, IL-15, and 4-1BB signaling promote precursor survival and Tpex differentiation (115, 158–161). Sustained cDC1–Tpex interactions within tdLNs help maintain high-avidity progenitor populations (108, 162).

Clinical translation faces practical constraints, as no trials have tested nodal-directed cytokine delivery, and systemic cytokine administration may lack nodal specificity and introduce toxicity.

### 6.3 Leveraging tdLNs for ACT

ACT exploits nodal T cell reservoirs. Conventional tumor-infiltrating lymphocyte products are enriched for terminally exhausted cells with limited proliferative potential (163). In contrast, tdLNs contain less differentiated Tpex populations with superior expansion capacity, higher Tpex frequency, broader TCR diversity, and preserved responsiveness to homeostatic cytokines (164).

Early-phase studies suggest feasibility of using sentinel node–derived lymphocytes in solid tumors (164) with ongoing clinical trial (ClinicalTrials.gov identifier: NCT05981001) currently evaluating this approach. Translation remains limited by metastatic node remodeling, including suppressive Treg–myeloid circuits and impaired antigen-presenting programs (13, 123, 124, 134).

Translation requires state-aware selection: enrichment for precursor-associated phenotypes (TCF1^+^, CD127^+^); supportive cytokine conditions (IL-7, IL-15) during ex vivo expansion; TCR-seq–based quality control confirming clonal diversity (129, 133); exclusion of metastatic or heavily remodeled nodes based on imaging or biopsy.

### 6.4 Biomarker-guided assessment and treatment planning for nodal function

Stratifying nodal state requires validated biomarkers reflecting antigen-presenting competence, precursor abundance, stromal integrity, and metabolic exposure—informing surgical timing and radiation field design.

Imaging-based approaches include PET metabolic activity, MRI-based assessment of high endothelial venules, and ultrasound elastography as indicators of nodal structural integrity (23). Tissue-based markers include TCF1^+^ CD8/T cell ratios, PD-L1^+^ DC frequency, FRC network integrity, and CD73 expression on lymphatic endothelial cells (6, 45, 56, 152). Blood-based exploratory markers include circulating Tpex-like cells, exosomal stromal signatures, and serum kynurenine/tryptophan ratios reflecting immunoregulatory activity in tdLNs (4, 5, 87).

Prospective validation studies linking nodal biomarkers to ICB outcomes remain limited but are essential for clinical translation. Beyond diagnostic stratification, nodal readouts can inform treatment planning. In radiotherapy, tdLNs can be treated as immune-relevant structures rather than default elective targets: dose–volume exposure of tdLNs has been linked to diminished lymphocyte activation and worse outcomes in clinical analyses, while preclinical data indicate that sparing or delaying tdLN irradiation better preserves synergy with ICB. When oncologically appropriate, integrating tdLN identification (e.g., via lymphatic mapping or anatomical drainage patterns) into planning may help balance regional control with preservation of immune renewal capacity (132).

## 7 Conclusions and future directions

tdLNs occupy a central yet underappreciated position in the regulation of antitumor immunity. Rather than functioning solely as conduits for metastatic spread, tdLNs act as dynamic immunological hubs in which T-cell fate decisions are established, precursor reservoirs are maintained or depleted, and therapeutic responsiveness is preconfigured prior to clinical intervention. Through coordinated structural remodeling, cellular reprogramming, and metabolic conditioning, tdLNs progressively reshape immune organization and determine whether Tpex populations retain the capacity for sustained clonal renewal during ICB. Within this framework, immunotherapy resistance cannot be fully explained by tumor-intrinsic mechanisms and may instead reflect upstream dysfunction within the lymph node compartment.

Despite these advances, several key translational gaps remain. Robust, clinically deployable biomarkers that capture tdLN immune fitness—including antigen-presenting competence, stromal niche integrity, precursor reservoir preservation, and immunoregulatory tone—are still limited and incompletely validated. Most available human data are cross-sectional and frequently obtained after therapeutic intervention, constraining reconstruction of temporal remodeling trajectories and limiting assessment of reversibility. In addition, direct, spatially resolved quantification of immunoregulatory metabolites within human tdLNs remains technically challenging, hindering the definition of actionable metabolic thresholds that govern immune priming versus tolerance. Prospective interventional studies evaluating tdLN-preserving or tdLN-targeted strategies, such as nodal-sparing radiotherapy, optimized surgical timing in neoadjuvant settings, or direct immune reprogramming of nodal compartments, remain scarce, and the balance between oncologic control and immune preservation is still context dependent.

Collectively, these limitations underscore a necessary shift from tumor-centric paradigms toward strategies that explicitly preserve and restore nodal immune competence. Intervention timing, preservation of stromal architecture, maintenance of trafficking pathways, and protection of Tpex reservoirs may be as critical as modulation of the tumor microenvironment itself. Future progress will depend on integrated spatial and longitudinal immune profiling linked to clinical outcomes, enabling stratification of tdLN functional states, development of state-aware biomarkers, and identification of patients most likely to benefit from nodal preservation or targeted reprogramming. As multi-omic and spatial technologies continue to refine our understanding of lymph node immune organization, cancer immunotherapy is likely to be increasingly guided not only by tumor-intrinsic features, but also by the functional integrity of the upstream immune infrastructure that sustains durable systemic antitumor responses.
